# Provider Discussion, Education, and Question-Asking about Control Medications during Pediatric Asthma Visits

**DOI:** 10.1155/2011/212160

**Published:** 2011-08-10

**Authors:** Betsy Sleath, Delesha M. Carpenter, Guadalupe X. Ayala, Dennis Williams, Stephanie Davis, Gail Tudor, Karin Yeatts, Chris Gillette

**Affiliations:** ^1^The University of North Carolina at Chapel Hill Eshelman School of Pharmacy, Chapel Hill, NC, USA; ^2^Cecil G. Sheps Center for Health Services Research, University of North Carolina at Chapel Hill, CB No. 7590, Chapel Hill, NC 27599-7590, USA; ^3^Division of Health Promotion and Behavioral Science, Graduate School of Public Health, San Diego State University, San Dieo, CA 92182-4162, USA; ^4^The University of North Carolina at Chapel Hill School of Medicine, Chapel Hill, NC 27599-7217, USA; ^5^Department of Science and Mathematics, Husson University, Bangor, ME, USA; ^6^Department of Epidemiology, The University of North Carolina at Chapel Hill, Chapel Hill, NC 27599-7435, USA

## Abstract

*Background*. Few studies have explored how providers communicate about control medications during pediatric asthma visits. *Objectives*. The purpose of this study was to: (a) describe the extent to which providers discuss, educate, and ask children and their caregivers questions about control medications and (b) examine how child, caregiver, and provider characteristics are associated with provider communication about control medications during pediatric asthma visits. *Methods*. Children ages 8 through 16 with mild, moderate, or severe persistent asthma and their caregivers were recruited at five pediatric practices in nonurban areas of North Carolina. After audio-tape recording medical visits, caregivers completed questionnaires and children were interviewed. Generalized estimating equations were used to analyze the data. *Results*. Providers educated families about control medications during 61% of the visits, and they asked questions about control medications during 67% of visits. Providers were significantly more likely to discuss control medications if a child was taking a control medication, if the child had moderate to severe persistent asthma, and if the child was present for an asthma-related visit. *Conclusion*. Providers need to educate and ask more questions of families about side effects and how well control medications are working.

## 1. Introduction

Asthma affects an estimated 8.9% of USA children under 17 years and continues to be one of the most common childhood chronic illnesses [1]. Uncontrolled asthma is associated with more school days missed among children, more work days missed among caregivers, and poorer quality of life among both [2, 3]. A special case of poor asthma control, nighttime awakenings from asthma, have been linked to school absences, lower school performance, and parents' lost workdays [4]. One factor that explains uncontrolled asthma is poor medication adherence [5]. 

The National Heart, Lung, and Blood Institute (NHLBI) guidelines provide several recommendations for proper asthma management to minimize uncontrolled asthma. These guidelines include: use of pharmacologic therapy, patient education, reduce environmental triggers, and assess and monitor asthma control [6]. The guidelines emphasize the importance of using a collaborative approach between providers, parents, and children to develop an appropriate asthma management plan for the child. However, recent studies have found that these guidelines are not being met, with less than half of families ever receiving any education about their child's asthma [7, 8]. In another study, most hospitalizations for asthma attacks were found to be preventable and had medications been taken regularly [9]. Prior research indicates that asthma patients who report poor communication with their physicians are less adherent with inhaled steroids [10, 11].

Few studies have examined how physicians communicate about medications during medical visits using actual communication data and to our knowledge, no prior study has investigated how physicians communication about control medications during pediatric asthma visits [12–15]. Findings from these prior adult studies suggest that communication about medications can be improved. In a sample of 40 Veterans who were on continued or newly prescribed antidepressants, providers asked 6% of patients about adverse events and 15% of patients how well the antidepressants were working. Moreover, providers only gave 10% of patients' information about adverse events and 5% information on how well the medication works [13]. Young et al. [14] used standardized patients (*n* = 131) and found that physicians provided information about side effects to 85% of patients but only gave information about how well the drug works to 38% of patients.

To our knowledge, there are no studies that have examined how providers communicate about control medications during pediatric asthma visits. It is important to better understand how providers communicate about control medications during medical visits because the clinical practice guidelines of the National Asthma Education and Prevention Program of NHLBI encourage physicians to discuss medications with patients at every follow-up asthma visit [6]. Therefore, the purpose of this study was to: (a) describe the extent to which providers discuss, educate, and ask children and their caregivers questions about control medications and (b) examine how child, caregiver, and provider characteristics are associated with provider communication about control medications during pediatric asthma visits. 

## 2. Methods

### 2.1. Participants

The study was approved by the University of North Carolina Institutional Review Board. Providers were recruited at five pediatric practices in nonurban areas of North Carolina, and consent was obtained. Children and their caregivers of these participating providers were recruited. Children were eligible if they: (a) were ages 8 through 16 years, (b) were able to speak English, (c) could read the assent form, (d) had been seen at the clinic at least once before, (e) were present at the visit with an adult caregiver (parent or legal guardian) who could read and speak English and who was at least 18 years of age, and (f) had mild, moderate, or severe persistent asthma. Persistent asthma was defined as experiencing asthma-related daytime symptoms more than twice a week, asthma-related nighttime symptoms more than twice a month, or receiving one or more long-term control therapies for asthma [16, 17].

Clinic staff referred potentially eligible and interested patients to a research assistant who explained the study, obtained caregiver consent and child assent, and administered the eligibility screener. Providers and families were told that the study was examining communication during pediatric visits. All of the medical visits were audio-tape recorded. Children were interviewed after their medical visits. Caregivers completed self-administered questionnaires. 

### 2.2. Audio-Tape Coding

All of the medical visit audio-tapes were transcribed verbatim, and a detailed coding tool was developed to assess provider communication behaviors. This tool was refined and tested over a one-year period. All of the transcripts were coded using the coding tool and more detail about the types of communication behaviors coded is provided below. 

### 2.3. Measures

#### 2.3.1. Demographic and Sociodemographic Characteristics

Medication use was assessed on the caregiver screener. The research assistants showed caregivers a list of asthma medications and asked them to indicate which one(s) the child was taking. Responses were dichotomized based on whether the caregiver reported that the child was taking a control medication versus not taking a control medication. Asthma severity was classified as mild versus moderate/severe by a research assistant based on recent symptoms and medication use reported by the caregivers when research assistants administered the eligibility screening instrument for the study [16, 17]. Our eligibility screening instrument utilized the primary asthma severity classification system that was being used when the study was designed and conducted [16, 17]. 

 All child study information was then reviewed by a pediatric pulmonologist or a clinical pharmacist with expertise in asthma to verify the severity classification as mild or moderate/severe persistent asthma. Severity was classified using two different methods. The first method was medication use; any child receiving a single long-term control agent was considered to have mild persistent asthma. Any child receiving two or more long-term control agents was categorized as having moderate to severe persistent asthma. A long-term control medicine included inhaled corticosteroids, leukotriene modifiers, cromolyn, nedocromil, or a long-acting beta agonist as defined by the National Heart Lung and Blood Insititute's guidelines [16, 17]. 

 The second method classified severity based on symptom frequency. Subjects who reported the occurrence of any one of eight symptoms as occurring two or more times per week or who reported awakening with asthma symptoms two or more times per month was classified as mild persistent. The eight daytime symptoms included: wheezing with a cold, wheezing without a cold, attack of wheezing that made it hard to breath or catch breath that lasted longer than a day or more, had a cough that would not go away, complained that chest felt tight or heavy, used rescue inhaler for symptoms, wheezed with exercise or running or playing hard, and coughed with exercise or running or playing hard. The nighttime symptoms asked about how often the child's sleep has been disturbed because of wheezing, coughing, chest tightness, or shortness of breath. Reports of daily symptom occurrence or awakening ≥5 times a month resulted in a classification as moderate or severe persistent. In situations where the two methods (medication use and symptom frequency) resulted in discordant classification, the more severe category was used. 

A variety of demographic factors were examined as potential confounders. Child and caregiver age, caregiver education, and years the child had asthma were measured as continuous variables. Child and caregiver gender were also measured. For descriptive purposes, child race was recoded into four categories: White, African American, Native American/American Indian, or Other (includes categories of: Hispanic, Asian American, other). However, for the bivariate analyses, child race was recoded into a dichotomous variable (White versus non-White). The child's insurance status was measured using the following categories: none, private insurance, Medicaid, the State Children's Health Insurance Program (SCHIP), and others. How well the child thinks the provider knows them as a person was measured with the following categories: hardly at all, slightly, moderately well, and very well. Reason for visit was measured as asthma-related versus other (e.g. physical). Length of visit was measured in minutes, and whether the child was taking a control medication was measured as a dichotomous variable. 

#### 2.3.2. Provider Discussion, Education, and Question-Asking about Control Medications

All of the medical visits audio-tapes were transcribed verbatim. A detailed coding tool was developed over a one-year period. The categories used in the coding tool for communication about asthma medications were adapted from the categories used in prior studies of provider-patient communication about medications [12–15]. The transcripts were reviewed by two research assistants who met twice a month with the investigators to develop and refine the coding rules until themes were saturated. 

Using the coding tool for transcribed medical visits, coders recorded the following: was there any discussion of control medications, did the provider give any education about control medications, and how many questions did the provider ask the child and caregiver about control medications. The research assistants then coded whether discussion, education, and question-asking occurred in each of the following areas: adherence, fears/concerns, frequency/timing, generic/brand, how well it works, purpose, side effects, strength/dose, supply, and others. Two research assistants coded 20 of the same transcripts throughout the study period to assess intercoder reliability which was calculated using interrater correlations. Inter-rater reliability was 1.0 for whether control medications were discussed, 0.87 for the number of areas discussed, 0.91 for whether the provider educated about control medications, 0.80 for number of areas the provider educated about, and 0.95 for the number of questions the provider asked about control medications. 

#### 2.3.3. Statistical Analysis

All analyses were conducted using SPSS v. 14. All children were included in the analyses because even if children were not on a control medication, control medications could have been discussed during the visit as a possible treatment. First, we present descriptive statistics for the demographic, clinical, and provider communication variables. Second, we examine bivariate relationships between the demographic variables and provider communication variables using correlation coefficients, *t*-tests, or Pearson chi-square statistics, as appropriate. 

Next, we used generalized estimating equations (GEE) to examine how demographic and clinical characteristics of the child and caregiver were associated with: (a) whether the provider discusses control medications, (b) whether the provider educates about control medications, and (c) how many questions the provider asks about control medications. All generalized estimating equations were clustered on provider. A Poisson GEE was used to examine provider question-asking because the variable was skewed. 

## 3. Results

### 3.1. Sample Characteristics

The five participating clinics were all primary care pediatric practices. Forty-one providers agreed to participate in the study; two providers refused to participate for a participation rate of 95.3%. Providers completed a short demographic questionnaire after providing consent. Three hundred and thirty-three of the 377 families (88%) that approached the research assistant to learn more about the study agreed to participate in the study. Two-hundred and ninety six patients of the 333 participating patients (89%) had useable audio-tape data, and these patients were seen by 35 of the 41 providers who agreed to participate in the study. Four of the 35 providers were nurse practitioners or physician assistants, and they saw seventeen of the participating children. Fifty-one percent of the providers were female. Twenty-seven of the providers were White, two were American Indian, three were African American, one was Asian, and two classified their race as other. Providers ranged in age from 30 to 70 years (mean = 44.8 years, standard deviation = 9.4). 

Table 1 presents the child and caregiver demographic characteristics. Forty-six percent of the children were female. The average age of the children was 11 years. Approximately 62% of the children were White, 30% were African American, and 10% were Native American/American Indian. In terms of the child's asthma, caregivers reported that their children had asthma for an average of six years. Seventy-two percent of these children had moderate to severe persistent asthma. Only three families in the sample did not have health insurance. Eighty-three percent of patients were on a control medication. 

### 3.2. Provider Discussion, Education, and Question-Asking about Control Medications

#### 3.2.1. Control Medication Discussion

Providers discussed control medications during 83.4% of encounters. Providers discussed control medications during 87.4% of visits where children were taking control medications and during 63.8% of visits where children were not taking control medications. 

The average number of topic areas discussed was 2.92 (standard deviation = 2.0; range 0 to 8 areas).  Table 2 shows the control medication areas that providers discussed most often during the medical visits: (a) frequency/timing of use (63%), (b) supply of medication (50%), (c) strength/dose of medication (48%), (d) adherence (47%), and (e) purpose of the controller medication (34%). Side effects were only discussed during approximately 11% of encounters and fears/concerns were only discussed during 4.4% of encounters. 

In the bivariate results, control mediations were significantly more likely to be discussed if a child was on a control medication (Pearson chi-square = 16.1, *P* < 0.001), if the child had moderate/severe persistent asthma (Pearson chi-square = 12.8, *P* < 0.001), and if the child was present for an asthma-related visit (Pearson chi-square = 14.48, *P* < 0.001).

Table 3 presents the GEE results predicting provider discussion of control medications. Providers were significantly more likely to discuss control medications if a child was on a control medication and during visits with children who had moderate to severe persistent asthma compared to children with mild persistent asthma. Providers were also more likely to discuss control medications when the reason for visit was asthma-related versus not asthma-asthma-related. 

#### 3.2.2. Control Medication Education

Providers educated about control medications during 61.1% of encounters. The average number of areas educated about was 1.54 (standard deviation = 1.6; range 0 to 7 areas). As presented in Table 2, providers educated about control medications most often in the following areas: (a) frequency/timing of use (37%), (b) strength/dose (32%), and (c) purpose (30%). Providers educated about side effects during only 7% of encounters and how well it works during only 8% of encounters. Providers were significantly more likely to provide education about control medication side effects if a child was not on a control medication (Pearson chi-square = 5.1, *P* = 0.02).

 In the bivariate results, providers were more likely to educate about control medications if the child had moderate/severe persistent asthma (pearson chi-square = 13.3, *P* < 0.001), if the child was younger (*t*-test = −2.71, *P* = 0.007), and if the child was present for an asthma-related visit (Pearson chi-square = 12.9, *P* < 0.001).

Table 3 demonstrates which child and caregiver characteristics were associated with whether providers educated families about control medications. Providers were significantly more likely to educate about control medications during visits with children who had moderate to severe persistent asthma, younger children, and during visits that were primarily asthma-related. 

#### 3.2.3. Control Medication Question Asking

Providers asked one or more questions about control medications during 66.6% of encounters. The average number of questions asked was 2.68 (standard deviation = 3.1; range 0 to 16 questions). Providers were most likely to ask children and their caregivers questions about control medications in the following areas: (a) frequency/timing of use (38%), (b) adherence (37%), and (c) supply (29%). Providers only asked about side effects during 2% of encounters, fears/concerns during 1% of encounters, and how well the control medications were working during approximately 12% of encounters (Table 2).

 In the bivariate results, providers were more likely to ask control medication questions if the child was on a control medication (*t*-test = 3.5, *P* = 0.001) and if the child was younger (Pearson correlation coefficient = −0.12, *P* = 0.04).

Table 4 presents the Poisson GEE results predicting the number of questions providers asked about control medications. Providers asked more questions about control medications if a child was currently treated with a control medication. Providers also were more likely to ask younger children more questions about control medications than older children.

## 4. Discussion

Providers discussed the frequency of use, supply of medication, and strength/dose of medication with families most often, but they only discussed the purpose of the control medication during about one third of all visits and how well the medication works during about a quarter of all visits. Providers rarely discussed side effects and fears/concerns about control medications. According to clinical practice guidelines of the National Asthma Education and Prevention Program of NHLBI, it is important for providers to discuss these areas with patients [6]. 

The national clinical practice guidelines instruct providers to teach and reinforce the roles of control medications at every opportunity [6], yet providers in this study only educated children and their caregivers about control medications during about two-thirds of the visits. Providers need to give more education about control medications during pediatric asthma visits. It is especially critical for families to understand the purpose of asthma control medications so that they understand the difference between control and rescue medications. 

Providers rarely educated about side effects and how well the medications work even though according to the practice guidelines of the National Asthma Education and Prevention Program of NHLBI, providers should ask about specific side effects from control medications during routine asthma visits [6]. Our results provide evidence that discussion of asthma controller medication does not occur at every follow-up visit. 

 Given recent evidence that poorly controlled asthma (56%) is common among children receiving asthma care from community pediatricians [18], this study points to provider discussion and education as a key area for improvement. If children and their caregivers better understand what to expect when taking the medications, they might be more adherent. Better adherence could lead to improved asthma control [6] and fewer school days missed for children and reduced health care costs [2, 3]. Future work needs to assess the relationship between provider education about control medications and medication adherence and asthma control. 

Communication about control medications was not associated with any provider demographic characteristics. However, there were certain child characteristics that were associated with communication. Specifically, providers were more likely to educate younger children about control medications and they were more likely to ask younger children more questions about control medications. Perhaps this is because younger children are less likely to volunteer information about their use and experiences in using control medications. It is important to make sure that children of all ages understand how to use their control medications. Providers also were more likely to engage children with more severe asthma in discussions about controller medications.

Providers asked about adherence during less than 40% of the visits. It is important for providers to ask about child adherence to control medications so they can work with families to improve adherence and asthma control. Providers asked few questions about side effects and how well the control medications were working, which is similar to findings of medication communication in adults with other medical conditions [12, 13]. If providers want to detect and prevent problems with asthma control medication use and adherence, they should consider asking at least one open-ended question about how the medications are working and a second question about any side effects or barriers to use (e.g., “How are your asthma medications working?” and “What types of problems have you had with your medications?”).

The study's generalizability is limited in that it was conducted in five pediatric clinics in nonurban areas of North Carolina. Another limitation is that clinic staff referred potentially eligible patients to the research assistant; thus, we do not know how many referred patients chose not to talk with the research assistant. However, we could not ask the clinic staff to track these numbers because of the busyness of the clinic and our promise not to interrupt clinic flow. Providers and caregivers knew they were being recorded and may have changed their communication, but they did not know the study hypotheses. It is also important to note that we did not assess the level of control in this study based on the current NHLBI guidelines [6] because this study was initiated prior to the release of the new guidelines. Another limitation is that we did not include children ages 2–7 years and adolescents ages 17-18 years. Future research should examine provider discussion, education, and question-asking about control medications in these age groups. Despite its limitations, this study presents information on the extent to which providers discuss and educate children about control medications during pediatric medical visits. 

## Figures and Tables

**Table 1:** Child and caregiver demographic characteristics (*N* = 296).

	Percent (*N*)
Child Age	
Mean (SD) Range	11.1 (2.4) 8–16 years
Child Gender	
Male	53.7 (159)
Female	46.3 (137)
Child Race	
White	61.5 (182)
African American	30.1 (89)
Native American/American Indian	10.1 (30)
Other	6.1 (18)
Asthma Severity	
Mild persistent	28.0 (83)
Moderate/Severe persistent	72.0 (213)
Number of years living with asthma	
Mean (SD) Range	6.0 (3.9) 9–16 years
Caregiver relationship status	
Never	16.2 (48)
Married	57.8 (171)
Separated	9.5 (28)
Divorced	12.5 (37)
Widowed	3.0 (9)
Caregiver Age	
Mean (SD) Range	42.0 (8.4) 27–81 years
Caregiver Gender	
Male	14.2 (42)
Female	85.8 (253)
Caregiver Education in Years	
Mean (SD) Range	12.8 (2.5) 2–20 years
Reason for Visit	
Asthma related	51.4 (152)
Other	48.7 (144)
Insurance Type	
None	1.0 (3)
Private	26.4 (78)
Medicaid	51.7 (153)
State Children's Health Insurance Program	51.7 (153)
Other	2.7 (8)
Reason for Visit	
Asthma related	51.4 (152)
Non-asthma related	48.6 (144)

**Table 2:** Communication about control medications during pediatric asthma visits (*N* = 296).

	Any discussion	Any education	Any question asking
	Percent (*N*)	Percent (*N*)	Percent (*N*)
Adherence	46.6 (138)	16.9 (50)	37.2 (110)
Fears/Concerns	4.4 (13)	3.4 (10)	1.0 (3)
Frequency/Timing	63.2 (187)	37.5 (111)	38.2 (113)
Generic/Brand	0 (0)	0 (0)	0 (0)
How well it works	22.6 (67)	7.8 (23)	11.8 (35)
Purpose	34.1 (101)	30.1 (89)	1.7 (5)
Side effects	10.8 (32)	7.1 (21)	1.7 (5)
Strength/Dose	47.6 (141)	32.1 (95)	22.3 (66)
Supply	50.0 (140)	13.5 (40)	29.4 (87)
Other	13.5 (40)	3.4 (19)	10.1 (30)

**Table 3:** Generalized estimating equation results predicting provider discussion of and education about control medications (*N* = 296).

Independent variables	Any discussion	Any education
	OR (95% CI)	OR (95% CI)
Child's severity of asthma, moderate severe	2.87 (1.69, 4.87)***	2.30 (1.58, 3.34)***
Years living with asthma	1.00 (1.00, 1.00)	1.00 (1.00, 1.00)
Taking a control medication	3.35 (1.59, 7.08)**	1.23 (0.53, 2.87)
Child age in years	0.90 (0.75, 1.08)	0.87 (0.79, 0.96)**
Child gender, female	0.58 (0.30, 1.12)	0.85 (0.54, 1.33)
Child race, White	1.37 (0.54, 3.47)	1.16 (0.57, 2.36)
How well child feels provider knows them as a person	0.90 (0.60, 1.36)	0.92 (0.73, 1.17)
Caregiver years of education	1.04 (0.90, 1.22)	1.02 (0.92, 1.13)
Provider race, White	1.71 (0.51, 5.82)	1.36 (0.53, 3.47)
Provider age	0.99 (0.93, 1.04)	1.00 (0.96, 1.30)
Provider gender, female	0.85 (0.31, 2.35)	0.70 (0.27, 1.84)
Length of visit	1.00 (1.00, 1.00)	1.00 (1.00, 1.00)
Reason for visit, asthma related	3.66 (2.05, 6.53)***	2.33 (1.52, 3.58)***

**P < .01, ***P < .001.

**Table 4:** Poisson generalized estimating equation results predicting the number of questions providers ask about control medications (*N* = 296).

Independent variables	*β* (95% CI)
Child's severity of asthma, moderate severe	0.19 (−0.32, 0.70)
Years living with asthma	0.00 (−0.00, 0.00)
Taking a control medication	1.15 (0.22, 2.07)*
Child age in years	0.19 (0.10, 0.29)***
Child gender, female	0.39 (−0.12, 0.89)
Child race, White	−0.41 (−0.83, 0.01)
How well child feels provider knows them as a person	0.06 (−0.14, 0.25)
Caregiver years of education	0.01 (−0.10, 0.13)
Provider race, White	0.17 (−0.61, 0.95)
Provider age	−0.02 (−0.06, 0.01)
Provider gender, female	−0.36 (−1.02, 0.30)
Length of visit	0.00 (−0.00, 0.00)
Reason for visit, asthma related	0.00 (−0.00, 0.00)

*P < .05, ***P < .001.
